# High Mobility Group Box 1 in Pig Amniotic Membrane Experimentally Infected with *E. coli* O55

**DOI:** 10.3390/biom11081146

**Published:** 2021-08-03

**Authors:** Igor Splichal, Alla Splichalova

**Affiliations:** Laboratory of Gnotobiology, Institute of Microbiology of the Czech Academy of Sciences, 54922 Novy Hradek, Czech Republic; splichal@gnotobio.cz

**Keywords:** high mobility group box 1, receptor for advanced glycation endproducts, Toll-like receptor, cytokines, amniotic membrane, amniotic fluid, intra-amniotic infection, preterm birth, pig

## Abstract

Intra-amniotic infections (IAI) are one of the reasons for preterm birth. High mobility group box 1 (HMGB1) is a nuclear protein with various physiological functions, including tissue healing. Its excessive extracellular release potentiates inflammatory reaction and can revert its action from beneficial to detrimental. We infected the amniotic fluid of a pig on the 80th day of gestation with 1 × 10^4^ colony forming units (CFUs) of *E. coli* O55 for 10 h, and evaluated the appearance of HMGB1, receptor for glycation endproducts (RAGE), and Toll-like receptor (TLR) 4 in the amniotic membrane and fluid. Sham-infected amniotic fluid served as a control. The expression and release of HMGB1 were evaluated by Real-Time PCR, immunofluorescence, immunohistochemistry, and ELISA. The infection downregulated HMGB1 mRNA expression in the amniotic membrane, changed the distribution of HMGB1 protein in the amniotic membrane, and increased its level in amniotic fluid. All RAGE mRNA, protein expression in the amniotic membrane, and soluble RAGE level in the amniotic fluid were downregulated. TLR4 mRNA and protein expression and soluble TLR4 were all upregulated. HMGB1 is a potential target for therapy to suppress the exaggerated inflammatory response. This controlled expression and release can, in some cases, prevent the preterm birth of vulnerable infants. Studies on suitable animal models can contribute to the development of appropriate therapy.

## 1. Introduction

Preterm birth (PTB) is birth before the completed 37 weeks of gestation and accounts for 75% of perinatal mortality and more than half the long-term morbidity [1]. PTB is associated with 5% to 18% of pregnancies and is caused by vascular disorders, decidual senescence, declined progesterone levels, cervical diseases, breakdown of maternal-fetal interference, stress, and infection [2]. Intra-amniotic infections (IAI) are presented in 10–15% of patients with preterm birth, and this ratio is increased to nearly 50% in very early preterm births [3]. Thus, the IAI plays a vital role in the birth of vulnerable preterm infants, and interventions to eliminate or reduce the appearance of the PTB have been studied. The mechanisms of the IAI that govern the PTB are not fully elucidated. It is known that the induction of inflammatory mediators, e.g., interleukin (IL) 1 and IL-6 [4], triggers it.

The experimental studies of the IAI leading to preterm birth are restricted in humans. Thus, different animal models are used to study preterm delivery. Non-human primates have the most similar reproductive biology to humans, and represent a near-ideal species to study the PTB [5], and the most frequently used non-human primate model is the rhesus monkey [6]. However, ethical constriction and high costs limit their use in experiments. Thus, other animal models such as mice, rats, rabbits, sheep [5], and pigs [7] are used in the IAI research. They differ from humans in the type of placentation [8], physiology of pregnancy, and mechanism to trigger labor and delivery [9] which results in translational limitations. A trophoblast is in direct contact with the mother’s blood in the human, but in the pig epithelial, connective, and endothelial tissues lie between trophoblast and mother’s blood [8]. These differences result in the passive immunization of the human fetuses via prenatal transfer of immunoglobulins, especially in the third trimester of the pregnancy [10]. In contrast, several tissue layers of the pig placenta create a barrier that prevents the prenatal transfer of high molecules such as immunoglobulins, and passive immunization of pig fetuses does not occur within the whole pregnancy. Piglets obtain their mother’s immunoglobulins and immune cells after birth via colostrum intake [11]. A similar situation is in the sheep syndesmochorial placenta [12] which belongs to another large animal model of the IAI [13,14].

Inflammation-induced labor can occur in the presence or absence of infection. In both cases, the innate immune reaction can be triggered by various ligands sensed by pattern recognition receptors (PRRs) [15]. These ligands can belong to microorganism-related pathogen-associated molecular patterns (PAMPs) and body-related damage-associated molecular patterns (DAMPs) [16,17]. PAMPs are, e.g., lipopolysaccharide (LPS), peptidoglycan (PG), and flagellin [17]. DAMPs are, e.g., high mobility group box 1 (HMGB1), urea crystals, and ATP [16]. DAMPs are sometimes called alarmins. They are endogenous intracellular factors commonly hidden from immune recognition. Under some circumstances, such as cellular stress or injury, they can be released to the cell vicinity and sensed [18]. HMGB1 is a molecule with multiple biological activities. It participates in various processes, e.g., transcription, replication, nucleosome formation, that occur in tissue healing, tissue remodeling, and other physiological processes. In contrast to many beneficial functions, it also engages in cancer, sepsis, miscarriages, and other morbidities [19,20,21]. HMGB1 can be produced by innate immune cells or released from cells undergoing necrosis [22]. Thus, circulating HMGB1 can arises from both active secretion and passive release [23] and can promote beneficial tissue repair in its physiological concentration, or its excessive levels provoke deleterious uncontrolled inflammation [24]. To date, at least 13 receptors for HMGB1 are known—RAGE, TLR2, TLR4, TLR9, NMDAR, CD24, TREM1, CXCR4, TIM-3, proteoglycans, integrin, α-synuclein, and IL-1R1 [25]. Among them, RAGE and TLR4 are considered the main receptors for HMGB1 [26]. Interaction of HMGB1 with its receptors triggers inflammatory pathways, e.g., NF-κB, p38, and ERK in cells in the vicinity of released HMGB1 [25].

*Escherichia coli* (*E. coli*) belong to -infectious agents that cause IAI [27]. *E. coli* O55 induced inflammatory cytokines IL-1β, Il-8, IL-18, and TNF-α in the *E. coli* O55 mono-associated piglets [28,29] and the infected amniotic fluid of the pig fetuses on 85th days of gestation [7,30]. In contrast, avirulent *E. coli* O86 did not show this effect, either in the gnotobiotic piglets [28,29], or pig amniotic fluids [7,30]. Thus, *E. coli* O55 was taken into experiments for its proven virulence.

Modulation of intra-amniotic inflammation could reduce the appearance of preterm births. The experimental infection of amniotic fluid with *E. coli* O55 aimed to evaluate: (i) the translocation of *E. coli* from the infected amniotic cavity to an adjacent one in the same uterine horn within 10 h of the experiment, (ii) the induction of HMGB1 transcription and expression on an amniotic membrane and its release into the amniotic fluid, and (iii) the induction of the HMGB1 receptors RAGE and TLR4, their transcription and expression in/on the amniotic membrane, and release into the amniotic fluid.

## 2. Materials and Methods

### 2.1. Bacterial Inoculum

Bacterial cultures of necrotoxigenic 2 *E. coli* O55 (O55:H-), in graphs abbreviated EcO55, were prepared for each experiment by growing the bacteria on meat peptone agar slopes (blood agar base; Oxoid, Basingstoke, UK) at 37 °C overnight. The bacteria were gently scraped off, resuspended in phosphate-buffered saline (PBS) pH 7.2, and diluted in log10 dilutions in saline (B. Braun Melsungen AG, Melsungen, Germany)**.** The number of colony-forming units (CFU) in the suspension was estimated by photometry at 600 nm. The CFU count was verified later by the cultivation on blood agar for 24 h at 37 °C.

### 2.2. Animals and Intra-Amniotic Infection

Miniature pregnant gilts free of common porcine transplacentally transmitted viral, bacterial, and protozoan pathogens (Animal Research Institute, Kostelec nad Orlici, Czechia) at gestation around 80th days were i.m. pre-medicated with 0.5 mg of atropine sulfate (Leciva, Prague, Czechia), 5 mg per kg of body weight of ketamine hydrochloride (Bioveta, Ivanovice na Hane, Czechia), and 2 mg per kg of body weight of azaperone (Janssen Pharmaceutica, Beerse, Belgium). Later, the gilts were anesthetized by inhalation of 1–2.5% of isoflurane (Nicholas Piramal Healthcare, London, UK) in combination with O_2_ and N_2_O (Linde, Prague, Czechia). The gilt was fixed in recumbency, laparotomy was performed, and the uterus was exteriorized. A three ml specimen of pre-infected amniotic fluid (AF) was aspirated through the uterine wall via 20G hypodermic needle for microbiological examinations, and 1 × 10^4^ CFUs of *E. coli* O55 in 3 mL of the apyrogenic saline (B. Braun Melsungen AG) was injected into the amniotic fluid. The bacterial inoculum was freshly prepared for each of the three experiments. A sham-infected control amniotic fluid was treated with saline only. The uterus was returned to the abdominal cavity, and an incision was sutured. The gilt was moved to a post-surgery unit and kept with free access to water till the surgery 10 h later. The gilts were again anesthetized, fixed on a surgery table, laparotomized, and amniotic fluids and membranes were taken. The samples were obtained from three independently performed experimental IAI. The used improved method was based on our former experiments [30,31].

### 2.3. Microbiological Examination of Pre-Infected Amniotic Fluids

The pre-infected amniotic fluids were checked for the possible microbial presence of aerobic and anaerobic bacteria, molds, and yeast, as described elsewhere. The specimens were cultivated for one week on broths (nutrient broth, Sabouraud broth, and Schaedler broth) and then on agars (blood agar, Sabouraud agar, and Schaedler agar). Smears of amniotic fluids were also stained using Gram method [32]. Mycoplasma test was performed on fixed Vero monkey kidney cell line using Hoechst 33258 staining (Sigma-Aldrich, St. Louis, MO, USA) according to the method described elsewhere [7]. The preparates were assessed under fluorescence microscope Olympus BX 40 microscope (Olympus, Tokyo, Japan).

### 2.4. Post-Infection Sampling

The amniotic fluids and amniotic membranes were collected 10 h post-infection via hysterotomy under the above-described anesthesia. The infected amniotic fluids were serially log10 diluted in PBS and aerobically incubated in 90 mm Petri dishes with MacConkey agar (Merck, Darmstadt, Germany) at 37 °C for 24 h and colonies were manually counted. The sham-infected amniotic fluids were cultivated undiluted. The amniotic fluids were spun for 15 min at 1500× *g* and 4 °C. The supernatants were after the addition of protease inhibitors (Roche Diagnostics, Darmstadt, Germany) aliquoted and stored at −40 °C till further processing.

### 2.5. Total RNA Purification and Reverse Transcription

A piece of approximately 1 mg of amniotic membrane stored in RNAlater was transferred to 350 μL of RLT Plus buffer 2 of RNAeasy Micro Plus kit (Qiagen, Hilden, Germany) in 2 mL Eppendorf tube with 2 mm diameter zirconia beads (BioSpec Products, Bartlesville, OK, USA) and homogenized in TissueLyser LT beadbeater (Qiagen) at 50 Hz for 5 min. Further steps of the total RNA purification followed the RNAeasy Micro Plus kit manufacturer’s instructions. The purity of the RNA was evaluated as a ratio of absorbances at 260 and 280 nm. Its quantity was measured by Quant-iT™ RiboGreen^®^ RNA Assay Kit (Life Technologies, Carlsbad, CA, USA) according to manufacturer’s instruction on the fluorescence microplate reader Infinite M200 (Tecan, Grödig, Austria). Ten ng of total RNA were reverse transcribed with the QuantiTect Reverse Transcription kit according to manufacturer’s instruction (Qiagen). Eighty μL of PCR grade water (Life Technologies) was added to 20 μL of the cDNA mixture to prepare a template for quantitative PCR.

### 2.6. Real-Time PCR

Two μL cDNA template was added to 18 μL of the FastStart Universal Probe Master (Roche Diagnostic, Manheim, Germany) containing 100 nM LNA probe (Roche Diagnostic), and 500 nM each of the forward and reverse primers (Generi-Biotech, Hradec Kralove, Czechia) to quantify specific sequences in the cDNA templates. The used LNA probe-based Real-Time PCR, and primers for HMGB1, RAGE, TLR2, TLR4, MyD88, and TRIF were listed elsewhere [33]. PCR was performed by the iQ5 Real-Time PCR cycler (Bio-Rad, Hercules, CA, USA) at 95 °C for 10 min (1×); 95 °C for 15 s; and 60 °C for 60 s (45×). GenEx Pro 6 software (Multid Analyses AB, Gothenburg, Sweden) was used for normalization of transcripts against β-actin and Cyclophilin A and for counting of their relative expressions.

### 2.7. Immunofluorescent Detection of HMGB1 in Amniotic Membrane

Amniotic membranes were embedded in Tissue-Tek (Sakura, Tokyo, Japan), snap-frozen in isopentane cooled in liquid nitrogen vapor and stored at −70 °C. Five μm acetone-fixed cryosections were cut on a cryostat CM1860 UV (Leica Microsystems, Wetzlar, Germany), put on SuperFrost/Plus slides (Thermo Fisher Scientific, Darmstadt, Germany), and were kept at −40 °C until labeling. Later, the sections were processed as described elsewhere [33]. Briefly, they were blocked with 10% normal rabbit serum (Life Technologies, Carlsbad, CA, USA) for 1 h at RT, incubated with anti-HMGB1 rabbit polyclonal antibodies (Novus Biologicals, Centennial, CO, USA) for 16 h at 4 °C, and labeled with Alexa Fluor 488-conjugated goat anti-rabbit IgG antibodies (Life Technologies) for 2 h at RT After embedding in ProLong Gold Antifade Reagent (Life Technologies), the sections were evaluated under an Olympus BX 40 microscope with an Olympus Camedia C-2000 digital camera (Olympus, Tokyo, Japan). The sections without primary antibodies served as controls. The HMGB1 and nuclei colocalization was verified by ImageJ software [34].

### 2.8. Immunochemical Detection of RAGE and TLR4 in the Amniotic Membrane

The prepared 5 μm cryosections were incubated with 3% H_2_O_2_ for 10 min and blocked with 10% normal rabbit serum (RAGE; Life Technologies, Carlsbad, CA, USA) or 10% normal goat serum (TLR4; Life Technologies) for 1 h at RT. Anti-human RAGE goat polyclonal antibodies (Bio-Rad, Hercules, CA, USA) or anti-human TLR4 rabbit polyclonal antibodies (Novus Biologicals, Centennial, CO, USA) labeled the sections overnight at 4 °C. After, they were incubated with a secondary antibody, peroxidase-conjugated F(ab)_2_ rabbit anti-goat IgG (RAGE; Jackson ImmunoResearch Europe, Ely, UK) or goat anti-rabbit IgG (TLR4; Life Technologies, Carlsbad, CA, USA) for 2 h at RT. The receptors were visualized by AEC substrate (Sigma-Aldrich, St. Louis, MO, USA) and nuclei were counterstained with Mayer’s hematoxylin (Diapath, Martinengo, Italy). The preparates were examined under an Olympus BX 40 microscope with Olympus Camedia C-2000 digital camera (Olympus, Tokyo, Japan). Control sections without primary antibodies were treated in the same way [35].

### 2.9. ELISA

After the sampling for bacterial cultivation, the amniotic fluids were spun at 1500× *g* for 10 min at 4 °C and supernatants were stored at −70 °C until processed. ELISA kits were used to measure HMGB1 (IBL Hamburg, Hamburg, Germany) and soluble receptors RAGE, TLR2, and TLR4 (all LSBio, Seattle, WA, USA). The values were measured in duplicates at 450 and 620 nm with the Multiskan RS Microplate Reader, and the results were evaluated by Genesis 3 software (both Labsystems, Helsinki, Finland).

### 2.10. Statistical Analysis

Kolmogorov–Smirnov’s test was used to evaluate the normality of distribution. An unpaired two-tailed Student t-test was used to compare values of non-infected and infected groups by GraphPad Prism 6 software (GraphPad Software, San Diego, CA, USA). The results of the measurements are presented in graphs as individual values (spots) with the mean as a horizontal line. The statistic differences between groups were considered non-significant (ns) or evaluated statistically significant *p* < 0.05 (*), *p* < 0.01 (**) or *p* < 0.001 (***).

## 3. Results

### 3.1. Survival of Infected Pig Fetuses and Translocation of E. coli O55

All sham-infected saline-treated piglets survived the 20 h experimental period, but their 1 × 10^4^ CFU of *E. coli* O55-infected counterparts showed 50% mortality. In contrast, all pig fetuses infected for 10 h survived. The injected 1 × 10^4^ CFU of *E. coli* O55 multiplied approximately 10^5^ times and showed 2.67 × 10^9^ (mean) ± 1.34 × 10^9^ (SEM) 10 h after injection, and no *E. coli* O55 was found in the amniotic fluid of the adjacent saline-treated (non-infected) amniotic cavity.

### 3.2. Expression of HMGB1, RAGE, TLR2, TLR4, MyD88, and TRIF mRNA in the Amniotic Membrane

**Figure 1:**
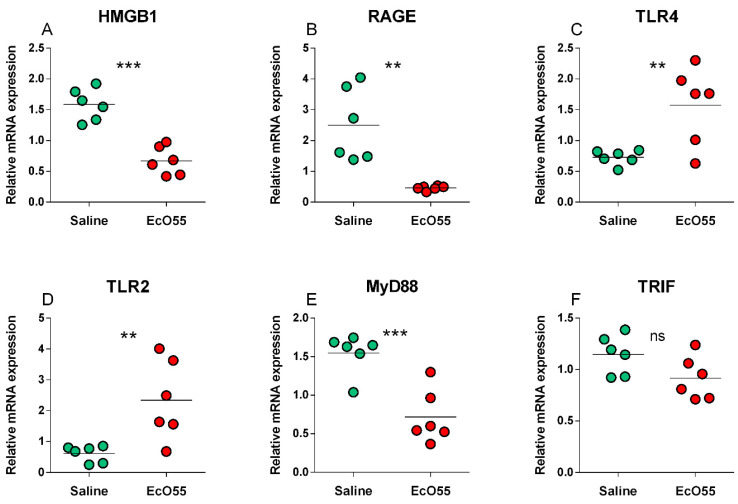
Relative mRNA expression (fold change) of HMGB1, RAGE, TLR2, TLR4, MyD88, and TRIF in the amniotic membrane. HMGB1 (**A**), RAGE (**B**), TLR4 (**C**), TLR2 (**D**), MyD88 (**E**), and TRIF (**F**) in the amniotic membrane of sham-infected (saline) and *E. coli* O55 (EcO55)-infected amniotic cavities. The individual values are depicted as green (saline) or red (*E. coli* O55) dots, and the horizontal line represents the mean. Statistical differences were calculated by the unpaired two-tailed Student t-test and labelled as non-significant (ns) or significant *p* < 0.05 (*), *p* < 0.01 (**), *p* < 0.001 (***). Six samples in each group were analyzed.

The expression of HMGB1 mRNA was statistically significantly higher (*p* < 0.001) in the non-infected amnion (Figure 1A). A similar trend, but with lower statistical significance (*p* < 0.01) showed the RAGE mRNA expression (Figure 1B). Opposite statistically significant trends showed TLR4 (*p* < 0.01; Figure 1C) and TLR2 (*p* < 0.01; Figure 1D) expression. MyD88 mRNA expression showed the same trend and significance (*p* < 0.001; Figure 1E) as HMGB1 mRNA (Figure 1A). No statistical differences between non-infected and infected amnions were found in TRIF mRNA expression (Figure 1F).

### 3.3. Colocalization of HMGB1 and Cell Nuclei in Amniotic Membrane

**Figure 2:**
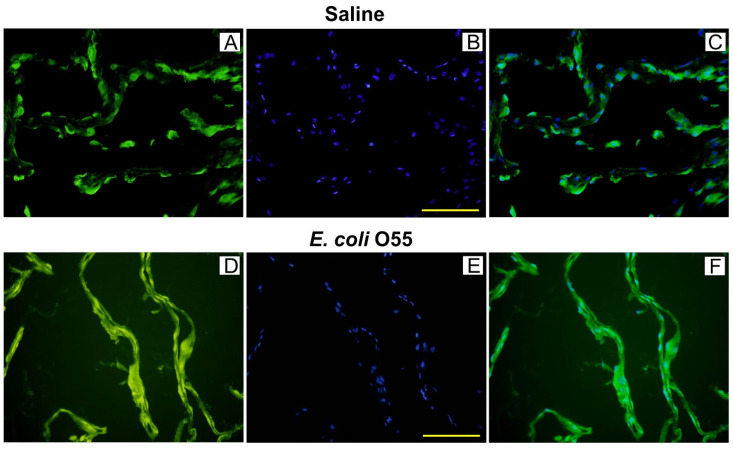
Colocalization of HMGB1 and cell nuclei in the amniotic membrane. HMGB1 (green; **A**,**D**), cell nuclei (blue; **B**,**E**), and their colocalization (**C**,**F**) are depicted on group representative micrographs. A bar (**B**,**E**) corresponds to 100 μm.

The expression of HMGB1 protein in the amniotic membrane with injected saline (Figure 2A–C) is localized in the cell nucleus and cytoplasm, as is shown in Figure 2C. In contrast, the amniotic membrane infected with *E. coli* O55 (Figure 2D–F) showed diffusely spread HMGB1 along the amniotic membrane.

### 3.4. Expression of RAGE in Amniotic Membrane

**Figure 3:**
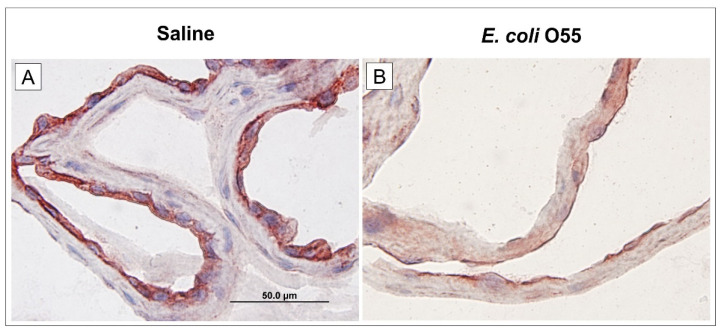
Expression of RAGE on amniotic membrane. Expression of RAGE in the amniotic membrane of the saline-treated (**A**) and *E. coli* O55-infected (**B**) amniotic membrane are depicted on group representative micrographs. A bar (**A**) corresponds to 50 μm.

The amniotic fluid infection with *E. coli* O55 downregulated the expression of RAGE on the amniotic membrane (Figure 3B) compared to the expression in the non-infected amniotic membrane (Figure 3A).

### 3.5. Expression of TLR4 in Amniotic Membrane

**Figure 4:**
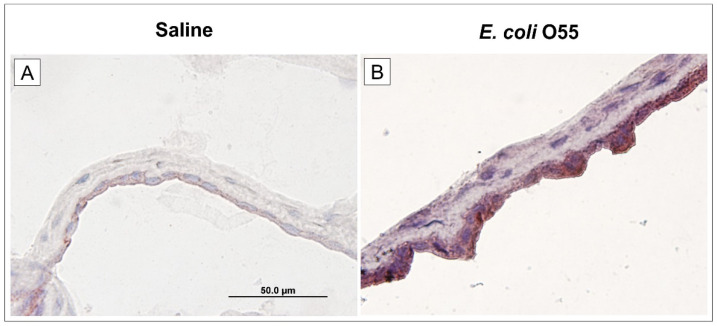
Expression of TLR4 on amniotic membrane. Expression of TLR4 in the amniotic membrane of the saline-treated (**A**) and *E. coli* O55-infected (**B**) amniotic membrane are depicted on group representative micrographs. A bar (**A**) corresponds to 50 μm.

The amniotic fluid infection with *E. coli* O55 upregulated the expression of TLR4 on the amniotic membrane (Figure 4B) compared to the expression in the non-infected amniotic membrane (Figure 4A).

### 3.6. Levels of HMGB1, sRAGE, sTLR2, and sTLR4 in the Amniotic Fluid

**Figure 5:**
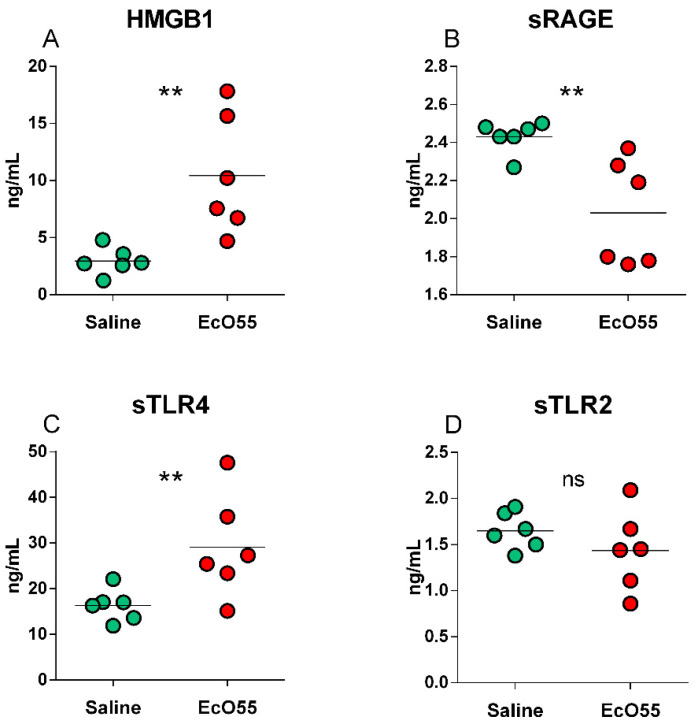
HMGB1, sRAGE, sTLR4, and sTLR2 levels in amniotic fluid. HMGB1 (**A**), sRAGE (**B**), sTLR4 (**C**), and sTLR2 (**D**) in amniotic membrane of sham-infected (saline) and *E. coli* O55 (EcO55)-infected amniotic cavities. The individual values are depicted as green (saline) or red (*E. coli* O55) dots, and the horizontal line represents the mean. Statistical differences were calculated by the unpaired two-tailed Student t-test and labelled as non-significant (ns) or significant *p* < 0.05 (*), *p* < 0.01 (**), *p* < 0.001 (***). Six samples in each group were analyzed.

HMGB1 was found statistically significantly higher (*p* < 0.01) in the *E. coli* O55-infected amniotic fluid (Figure 5A). The detected soluble receptors showed various relations between the saline-treated non-infectious control and *E. coli* O55-infected amniotic fluid (Figure 5B–D). sRAGE (Figure 5B) was statistically significantly lower (*p* < 0.01) in the infected amniotic fluid, sTLR4 (Figure 5C) statistically significantly higher (*p* < 0.01), and sTLR2 comparable between both groups (Figure 5D).

## 4. Discussion

Intra-amniotic infections (IAI) are one of the reasons for the preterm birth of vulnerable infants [2]. Alleviation of IAI impact can increase the length of gestation and postpone the preterm birth (PTB), which beneficially supports organ development, mainly of the lungs [36]. The possibility of the experimental work in the preterm infant is very limited, e.g., to a passive collection of various data, and experiments with other primates have ethical and financial limitations. Thus, other animal models are used to simulate IAI in humans [5,7,14]. In the experimental work, IAI inducing agents are viruses [37], mycoplasma [38], bacteria [7,39], or their immunomodulatory parts such as lipopolysaccharide (LPS) [40,41].

Intra-amniotic inflammation can be, according to its origin, divided into two basic groups: (i) infection and (ii) sterile inflammation. They share closed characteristics [2,21,42] and include both Janeway’s “stranger model” [15] and Matzinger’s “danger model” [43]. Thus, intra-amniotic inflammation can be provoked by infection with detectable infectious agents, or it can occur as a sterile inflammation with the absence of detectable microorganisms [44]. It implies that pro-inflammatory stimuli can result from infection (PAMPs) or tissue damage (DAMPs). HMGB1 participates in sepsis and sterile inflammation, and creates a bridge between these detrimental reactions if its release is exaggerated.

Intra-amniotic application of an HMGB1 induced preterm parturition in mice and high mortality of their pups within the first week of life. Intraperitoneal injection of HMGB1 did not show such effect [45]. Regarding the inflammatory reaction, HMGB1 mediates endotoxin shock [46], and can cause multiple organ dysfunction syndrome (MODS) [47]. It is “a late mediator of sepsis” with postponed release in comparison with “early mediators of sepsis” represented, e.g., by TNF-α [48]. Its excessive plasmatic levels were found in patients who suffered from severe sepsis [49]. Although HMGB1 was primarily described as the nuclear protein, it is possible to found it also in the cytosol, mitochondria, and membrane surface [19]. Released HMGB1 in the extracellular milieu conveys danger signals sensed by various receptors [25]. However, it is necessary to consider that each of these receptors do not recognize exclusively one ligand only, but each of them usually recognize multiple ligands [17,18,25,50,51]. We paid attention to *E. coli* O55 IAI-induced HMGB1, RAGE, and TLR4, and partially to TLR2, which is another representative of TLR that senses both bacterial patterns and HMGB1 [17,18]. Adaptor molecules MyD88 and TRIF served to describe the modulation of these signaling pathways in the porcine amnion in vivo.

In our experiments, the infection with *E. coli* O55 downregulated HMGB1 mRNA expression. Non-influenced expression of HMGB1 mRNA in the ileal epithelium, but high upregulation in the ileal content, were described in the gnotobiotic piglets infected with the same *E. coli* [28]. HMGB1 de novo synthesis, localization, migration, and biological activity are governed by its posttranslational modifications. Hyperacetylation [52], hyperphosphorylation [53], or mono-methylation [54] move HMGB1 from the nucleus to the cytoplasm. The final HMGB1 secretion triggers lysophosphatidylcholine that is later produced in the inflammation site [55]. The system of the post-translational modifications moves HMGB1 between cell compartments and releases it from cells. This may be a reason that we did not find activated mRNA expression within 10 h experimental period. After the exocytosis, the secreted HMGB1 can create complexes with its receptors [25]. RAGE and TLR4 are considered the main receptors for HMGB1 [26]. Thus, we focused our attention on them.

HMGB1 in women without IAI was localized in the nuclei and cytoplasm of amniotic epithelial cells. In the infected amnion, HMGB1 was spread in the cytoplasm [56]. We found that HMGB1 protein was colocalized in amnion epithelial cell nuclei and the cytoplasm in the saline-treated sham-infected control. In contrast, the *E. coli* O55-infected amnions showed extracellular localization of released HMGB1 that created a continuous diffuse line along the epithelial membrane. This appearance of HMGB1 in the infected amnion proposes cytokine disruption of tight junctions among adjacent amnion epithelial cells [57]. The infection with *E. coli* O55 increased HMGB1 levels in the amniotic fluid compared to the saline-treated amnions. Concordantly with our results, HMGB1 mRNA in sheep injected with LPS into amnion was not changed, but an expression of the protein was increased within 5 h [14]. This fast appearance contradicts the proposition of HMGB1 as the late mediator of sepsis inducing prenatal lethality in mice [45,48]. We found approximately three times increased levels of the infected amniotic fluid HMGB1 10 h after IAI. A similar ratio was reported in the case of amniotic fluids of women without chorioamnionitis versus patients with detected chorioamnionitis [58]. Other researchers found that amniotic fluid HMGB1 levels are not dependent on a gestational age, but were increased in women with IAI and PTB [59]. One of the possible sources of HMGB1 in the amniotic fluid may be the constitutive secretion by the amnion, which can be emphasized by its infection or injury [56,60]. In contrast to the relation of IAI and increased HMGB1 levels, it seems that infection-induced intra-amniotic inflammations are less frequent than sterile inflammation [61].

RAGE was initially described as a receptor for a heterogeneous group of non-enzymatically glycated proteins and lipids called advanced glycation endproducts. It can be highly upregulated in various pathological processes such as diabetes, cardiovascular diseases, and cancer [62]. HMGB1 mediates its pro-inflammatory effects through binding to RAGE and other receptors, e.g., TLR2 and TLR4, on various cell lines as monocytes, neutrophils, endothelial, and epithelial cells [63]. LPS (sometimes called endotoxin) is the major component of the cell wall of Gram-negative bacteria [64]. It can associate with RAGE directly, or via TLR4/MD-2 complex, and activate pro-inflammatory signaling. The RAGE associated with LPS can regulate inflammatory responses [17,65]. After binding the appropriate ligands, RAGE triggers various signaling pathways, including mitogen-activated protein (MAP) kinases (ERK1/2, p38, SAPK/JNK), STAT3, Akt, and Rho GTPases (Rac1, Cdc42) [51]. We found lower RAGE mRNA and protein expressions in the amniotic membrane and sRAGE in AF in the *E. coli* O55 infection case than the saline-treated one. As RAGE is a multi-ligand receptor that participates in various physiological and pathological processes [51,66], it is hard to eliminate multiple factors that can influence its expression.

Membrane-bound RAGE can be cleaved by matrix metalloproteinases and released from the cell surface. The released soluble RAGE (sRAGE) can regulate signal transduction [65]. The significant increase of the amniotic fluid HMGB1 concentration and a decreased amniotic fluid sRAGE in our experiment concur with findings observed in chorioamnionitis in women at term delivery [58]. However, these results contradict the findings in intra-amniotic infection/inflammation in preterm gestations [67]. Thus, it suggests that HMGB1 and sRAGE may regulate the inflammatory responses in both term and preterm gestations, and sRAGE can be used as a therapeutic tool, e.g., for LPS-induced septic shock [65]. In contrast, other researchers found that sRAGE AF levels in women did not influence the presence of the intra-amniotic inflammation with or without cultivated infectious agents [68]. In the ovine model of the IAI, neither RAGE mRNA, nor protein expressions were changed in fetal membranes 5 h–15 days after intra-amniotic application of LPS [14].

TLRs are involved in physiological regulations in many acute and chronic diseases [69]. TLR4 senses LPS and HMGB1, which represent PAMPs and DAMPs, respectively [18,70]. LPS released into blood circulation causes life-threatening endotoxin shock [46]. After its release, LPS creates a complex with lipopolysaccharide-binding protein (LBP). The LPS–LBP complex binds to a membrane-bound TLR4 coreceptor CD14 that is expressed mainly on phagocyting cells and enterocytes [71]. The secreted myeloid differentiation protein 2 (MD-2) in the TLR4/MD-2 complex is essential for the responsiveness of TLR4 to LPS [50,69]. Membrane-bound receptors and their coreceptors can be cleaved into their soluble forms and downregulate signaling [65,72,73]. TLR4 expressed in amnion epithelium senses amniotic fluid for the presence of pathogens [74]. Higher amniotic fluid sTLR4 was typical for microbial invasion of the amniotic cavity in women [75].

In our experiments, TLR4 was significantly upregulated in mRNA and protein levels by the infection and cleaved to sTLR4. The importance of TLR4 as a possible therapeutic IAI target was reported [76,77]. TLR4 is only one of the TLRs which use both myeloid differentiation factor 88 (MyD88) and TIR domain-containing adaptor protein inducing IFN-γ (TRIF) pathways for downstream signaling. The MyD88 and TRIF pathways are responsible for the production of pro-inflammatory cytokines and type I interferons, respectively. MyD88 signaling branch is used in TLR4 on the cellular membrane and TRIF signaling branch in internalized TLR4 [17,50]. Combined genetic disruption of TLR4 and MyD88 showed that the triggering of parturition is dependent on TLR4, but MyD88 did not influence it. TLR4 may trigger parturition by binding endogenous ligands and signaling via TRIF [78]. We found downregulation of MyD88 mRNA, but similar TRIF mRNA expression. Downregulated MyD88 mRNA can be a response to increased levels of sTLR4 and other components of its signaling pathway as CD14 and MD-2. They can serve as decoy receptors and regulate excessive stimulation. This can mean that other ligands than *E. coli* O55 LPS can participate in TLR4 upregulation.

TLR2 is usually considered the main TLR that recognizes Gram-positive bacteria. It recognizes peptidoglycan (PGN), lipoproteins, and lipoteichoic acid, but lipoteichoic acid is only typical for Gram-positive bacteria [50]. Overlapping recognition of “Gram-positive” by TLR2 and “Gram-negative” by TLR4 amplifies shared molecules of coreceptors CD14 and MD-2 [79], and MD-2 enabled TLR2 to respond to a broad range of LPS structures and lipoteichoic acid [80]. Gram-negative bacteria-induced innate response in conventional mice also neglected signals via TLR2 [81]. Upregulation of TLR2 in gnotobiotic piglets infected with Gram-negative *Salmonella* Typhimurium was described [33], and this upregulation was proportional to the completeness of LPS [35]. It is also necessary to consider that Gram-negative bacteria contain thin peptidoglycan that can be sensed by TLR2 [50]. Moreover, the infection with *E. coli* O55 upregulated the release of HMGB1 that belongs to the TLR2 ligands [18,82], and thus can also stimulate this signaling pathway. These facts can explain upregulated TLR2 mRNA in the infected amnion. In contrast, levels of sTLR2 in the infected and non-infected amniotic fluids were comparable. The presence of the truncated sTLR2 in the amniotic fluid was reported as constitutive, and its levels were dependent on gestational age [83]. The same group also described the constitutive presence of sMD-2 that was upregulated by IAI [72]. MD-2 participates in TLR4 and TLR2 sensing [70]. Soluble forms of TLR4 and TLR2 and their coreceptors MD-2 and CD14 act as decoy receptors/coreceptors and regulate the host inflammatory response to bacteria [72,73].

## 5. Conclusions

Intra-amniotic infections are one of the reasons for preterm birth. HMGB1 participates in various physiological processes as tissue healing, but its excessive release potentiates inflammatory reaction and can negatively impact the organism. The *E. coli* O55-infected pig amniotic fluids influenced HMGB1, RAGE, and TLR 4 expression in the amniotic membrane and released HMGB1, sRAGE, and sTLR4 into the amniotic fluid. Thus, the infection/inflammation-modified expression of HMGB1, RAGE, and TLR4 showed a therapeutic potential of these biomolecules. Future translational research on suitable animal models targeted to controlled expression and release of HMGB1, RAGE, and TLR4 is needed to develop an appropriate therapy to prevent preterm birth.

## Data Availability

Data are available on request from the corresponding author.
